# Anti-CD19 monoclonal antibodies for the treatment of relapsed or refractory B-cell malignancies: a narrative review with focus on diffuse large B-cell lymphoma

**DOI:** 10.1007/s00432-021-03833-x

**Published:** 2021-11-06

**Authors:** Pier Luigi Zinzani, Giorgio Minotti

**Affiliations:** 1 IRCCS Azienda Ospedaliero-Universitaria di Bologna, Istituto di Ematologia “Seràgnoli”, Via Massarenti 9, 40138 Bologna, Italy; 2 Department of Specialist, Diagnostic and Experimental Medicine, University of Bologna, Bologna, Italy; 3 Department of Medicine, Center for Integrated Research and Unit of Drug Science, University Campus Bio-Medico, Rome, Italy

**Keywords:** Anti-CD19, Non-Hodgkin lymphoma, Acute lymphoblastic leukaemia, Antibody–drug conjugate, B lymphocytes, Treatment resistance

## Abstract

**Purpose:**

CD19 is a cell surface protein that is found on both healthy and malignant B cells. Accordingly, it has become an important target for novel treatments for non-Hodgkin lymphomas and B-cell leukaemia. Three anti-CD19 monoclonal antibodies with distinct mechanisms of action have been developed for the treatment of B-cell malignancies.

**Methods:**

We reviewed the preclinical and clinical data on the development of the newly approved anti-CD19 monoclonal antibodies blinatumomab, tafasitamab and loncastuximab tesirine, and consider their place in the treatment of relapsed or refractory B-cell malignancies.

**Results:**

Blinatumomab is a bispecific T-cell engager that binds to both CD19 on B cells and CD3 on T cells, facilitating antibody-dependent cytotoxicity. Blinatumomab significantly prolongs overall survival in patients with relapsed or refractory B-cell acute lymphoblastic leukaemia, although cytokine release syndrome and severe neurotoxicity may necessitate discontinuation. Tafasitamab, which has modified anti-CD19 Fab and Fc regions, has significantly enhanced affinity for both CD19 and effector cell receptors compared with unmodified anti-CD19. In L-MIND, tafasitamab plus lenalidomide provided an overall response rate (ORR) of 57.5% in patients with relapsed or refractory diffuse large B-cell lymphoma (DLBCL) in patients non-transplant eligible. Loncastuximab tesirine is an antibody–drug conjugate that has been studied as monotherapy and in combination with ibrutinib in 3L + relapsed or refractory DLBCL. The ORR was 48.3% in a phase II trial of loncastuximab tesirine. The optimal place of anti-CD19 monoclonal antibodies in therapy has yet to be determined, but the prospect of improved outcomes for at least some patients with treatment-resistant B-cell malignancies appears likely, particularly in those with limited therapeutic options and poor prognosis.

## Introduction

The lymphomas are a heterogeneous group of malignant diseases caused by the clonal proliferation of lymphocytes. Histologically, they are classified as Hodgkin or non-Hodgkin lymphomas (NHL), with the latter accounting for 90% of cases (Jamil et al. 2021). NHL is largely a disease of later life, with the median age at diagnosis being 67 years (Sapkota et al. 2020). Diffuse large B-cell lymphoma (DLBCL) is the most common type of NHL, accounting for 25–30% of cases (Jamil et al. 2021). An aggressive lymphoma with a high mortality rate (Camicia et al. 2015), DLBCL is characterized by large lymphoid cells that carry B-cell surface antigens such as CD19 and CD20 (Jamil et al. 2021).

The monoclonal antibody rituximab is directed against CD20. With US Food and Drug Administration (FDA) approval in 1997, it became the first biological to be licensed for the treatment of a B-cell malignancy (Horst et al. 2020). Since then, the addition of rituximab to the CHOP regimen (cyclophosphamide, doxorubicin, vincristine and prednisone) has significantly improved response rates and survival in patients with newly diagnosed DLBCL (Tilly et al. 2015; Coiffier et al. 2002). Approximately 60% of patients achieve remission with R-CHOP; a further 30–40% experience a disease relapse, while 10% have refractory disease (Gisselbrecht and Neste 2018). In patients with relapsed or refractory disease, the standard second-line treatment is salvage chemotherapy followed by autologous stem cell transplantation (ASCT); however, 50% of patients who receive salvage chemotherapy will have an inadequate response and thus be ineligible for ASCT, while a further 25% will relapse following ASCT (Gisselbrecht and Neste 2018). Thus, only around 25% of these patients will derive long-term benefit. Prognosis in this patient population is poor (Crump et al. 2017), and the relative lack of effective subsequent options continues to be one of the main unmet needs in the field of haemato-oncology (Harris et al. 2020).

The success of rituximab has helped establish immunotherapy, and monoclonal antibodies in particular, as an integral part of anticancer therapy (Horst et al. 2020). Monoclonal antibodies exert their anticancer effects by activating cytotoxic pathways, including antibody-dependent cellular cytotoxicity (ADCC), antibody-dependent cellular phagocytosis (ADCP), and complement-dependent cytotoxicity (CDC) (Horst et al. 2020; Abramson et al. 2020). Direct effects are also possible: for example, receptor blockade may cause cell death by diminishing downstream growth signals. However, as mentioned above, a substantial proportion of patients with DLBCL experience early relapse following rituximab-based therapy, or are refractory to it. This has prompted the development of monoclonal antibodies targeting B-cell antigens other than CD20. In addition, considerable effort has been devoted to the development of optimised monoclonal antibodies with enhanced cytotoxic effector functions (Katz and Herishanu 2014).

In addition, CD19 has attracted considerable interest as a potential target to treat B-cell malignancies (Katz and Herishanu 2014; Zalevsky et al. 2009). Several treatments that target cancer cells via CD19 have either entered the market or are undergoing regulatory evaluation, including modified monoclonal antibodies against CD19 (e.g. tafasitamab) and chimeric antigen receptor (CAR) T-cell therapies (e.g. tisagenlecleucel). Compared with CAR T-cell therapies, which at present are resource-intensive in terms of implementation, manufacturing time, and costs (Harris et al. 2020; Abramson et al. 2020; Patriarca and Gaidano 2021), anti-CD19 monoclonal antibodies will likely be accessible to a greater proportion of patients with B-cell malignancies.

The aim of this review is to present and summarize our current knowledge on the newly approved anti-CD19 monoclonal antibodies blinatumomab, tafasitamab and loncastuximab tesirine, and to consider their place in the treatment of relapsed or refractory B-cell malignancies.

## Rationale for targeting CD19 in B-cell malignancies

CD19 is a transmembrane receptor that is specific for the B-cell lineage (Katz and Herishanu 2014). Unlike CD20, CD19 is expressed throughout the entire B-cell maturation process (Katz and Herishanu 2014), and studies in mice lacking CD19 have demonstrated that it has an important role in B-cell maturation and activation (Zalevsky et al. 2009; Poe et al. 2012). CD19 has thus become an interesting target for the immunotherapy of NHLs and B-cell leukaemias (Anderson et al. 1984; Ginaldi et al. 1998; Olejniczak et al. 2006; Narkhede and Tafasitamab 2020). Although levels of expression are lower for CD19 than CD20, CD19 is expressed by a broader spectrum of lymphoid malignancies due to its presence from the early stages of B-cell development (Zalevsky et al. 2009; Anderson et al. 1984; Horton et al. 2008).

## Development and optimisation of anti-CD19 antibodies

Over the past three decades, several anti-CD19 monoclonal antibodies have been evaluated for the treatment of B-cell malignancies (Katz and Herishanu 2014; Zalevsky et al. 2009; Narkhede and Tafasitamab 2020; Roßkopf et al. 2020). Initial results with unmodified or drug-conjugated anti-CD19 antibodies were modest, and research efforts subsequently focused on antibody optimisation and strategies to potentiate immune effector cell activity (Horst et al. 2020; Katz and Herishanu 2014; Narkhede and Tafasitamab 2020). Currently available drugs contain humanized anti-CD19 that has undergone modification to potentiate its anticancer activity (Katz and Herishanu 2014; Narkhede and Tafasitamab 2020). Three different modification strategies have been implemented, leading to three different types of anti-CD19 antibodies: bispecific T-cell engagers (BiTEs; e.g. blinatumomab), fragment crystallizable (Fc)–engineered and Fab affinity-matured antibodies (e.g. tafasitamab [MOR208]), and new-generation antibody–drug conjugates (e.g. loncastuximab tesirine [ADCT-402]) (Katz and Herishanu 2014; Narkhede and Tafasitamab 2020).

## Bispecific T-cell engagers

**Fig. 1:**
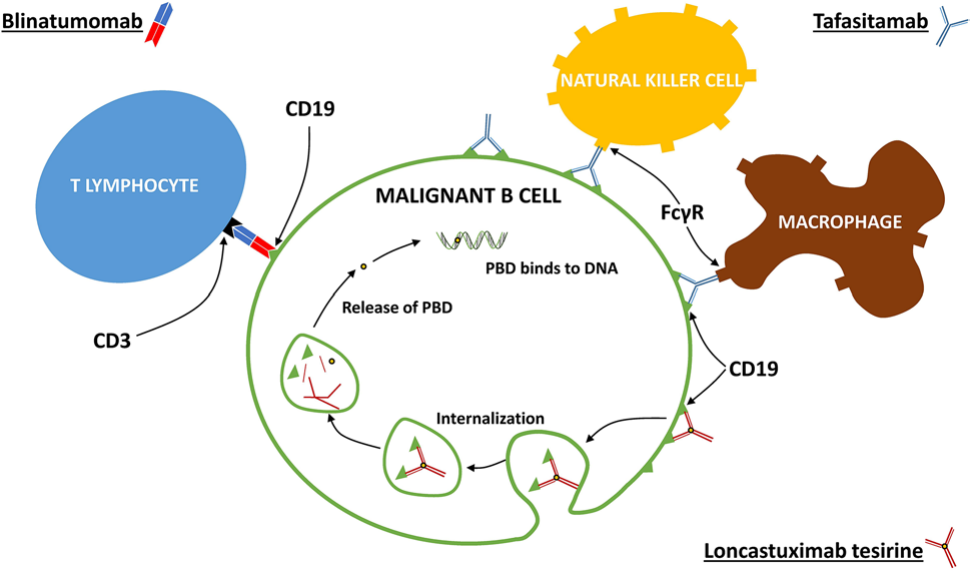
Anti-cancer mechanism of action of antibodies directed against CD19 on the surface of malignant B cells (Hartley 2020; Duell et al. 2019; Cheson et al. 2021). Blinatumomab (top left) binds simultaneously to CD19 and to CD3 receptors on T-cells, which brings the effector and malignant cells into close proximity to each other, and facilitates antibody-dependent cellular cytotoxicity (ADCC). Tafasitamab (top right) binds with high affinity to both CD19 and Fc gamma receptors (FcγR) on effector cells. Binding to FcγRIII on natural killer cells facilitates ADCC, while binding to FcγR on macrophages facilitates antibody-dependent cellular phagocytosis (ADCP). Tafasitamab also has direct cytotoxic effects. Loncastuximab tesirine (bottom right) is an anti-CD19 antibody–drug conjugate that contains a cytotoxic pyrrolobenzodiazepine dimer (PBD). Antibody binding to cell surface CD19 leads to internalization of the complex and intracellular release of the PBD payload. The PBD then binds to the minor groove of DNA, and forms inter-strand cross-links that are not recognized by DNA repair mechanisms, thereby leading to cell death

BiTEs are able to bind to two antigens simultaneously: typically, one antigen on a malignant cell, and the other on a T cell. This brings malignant cells and effector cells into close proximity to each other, facilitating ADCC (Fig. 1) (Abramson et al. 2020; Katz and Herishanu 2014). Blinatumomab was the first BiTE antibody to be approved; it binds to CD19 on malignant B cells, and to CD3 on T cells (Abramson et al. 2020). The two binding sites are connected by a linker (Abramson et al. 2020).

## Fc-engineered and Fab affinity-matured antibodies

The cytotoxic effects of monoclonal antibodies are mediated by their Fc regions, which are therefore critical for therapeutic efficacy (Horst et al. 2020). Engineering of the Fc domain, via modification of the Fc glycosylation profile (glyco-engineering) or site-directed mutagenesis of the Fc domain (protein engineering), is a novel approach to antibody modification (Horst et al. 2020; Narkhede and Tafasitamab 2020; Roßkopf et al. 2020; Kellner et al. 2017). ADCC and ADCP responses are induced when the Fc domain binds to Fc gamma receptors (FcγR) that are expressed on a variety of effector cells, including natural killer (NK) cells and macrophages (Horst et al. 2020). NK cells, which express only the FcγRIIIa member of the FcγR family,^1^ are the most potent inducers of ADCC (Horst et al. 2020), while macrophages mediate ADCP.

Tafasitamab, the first Fc-engineered monoclonal antibody to be approved for the treatment of DLBCL, has two amino acid mutations in the Fc domain (S239D and I332E) that, remarkably, result in a ≥ 40-fold increase in its affinity for FcγR, including FcγRIIIa (Horst et al. 2020; Narkhede and Tafasitamab 2020; Horton et al. 2008). At the same time, humanization and affinity maturation of the variable region (Fab) nearly doubles the affinity of tafasitamab for CD19 compared with its immunoglobulin G precursors (Horton et al. 2008). Augmented affinity for both effector and target cells bestows functional advantages upon tafasitamab that are reminiscent of a bispecific antibody; as a result of these modifications, tafasitamab is associated with a 10- to 1000-fold increase in ADCC compared with an unmodified analogue.

Tafasitamab was optimized using a proprietary technology that uses a novel method of variable fragment (Fv) humanization to maximize the human sequence content and enhance affinity for antigen and for effector cell FcγRs. As a result of this process, tafasitamab can be considered an enhanced next-generation anti-CD19 monoclonal antibody. Its mechanism of action is summarized in Fig. 1.

## Antibody–drug conjugates

Antibody–drug conjugates combine selectivity and cytotoxic potency because they facilitate the targeted delivery of cytotoxic agents to cancer cells via antibodies directed against tumour-associated cell surface antigens, such as CD19 (Kahl et al. 2019; Diamantis and Banerji 2016). They, therefore, have the potential to optimize efficacy while reducing systemic toxicity (Jain et al. 2020). Loncastuximab tesirine, an anti-CD19 antibody–drug conjugate, was approved by the FDA in April 2021 (US Food and Drug Administration. 2021a), and is undergoing evaluation by the European Medicines Agency (EMA). In loncastuximab tesirine, a humanized anti-CD19 antibody is stochastically conjugated to a cytotoxic pyrrolobenzodiazepine (PBD) dimer (Kahl et al. 2019). Following antibody-CD19 complex cellular internalization and sequestration in lysosomes, PBD is released and acts as a DNA minor-groove cross-linking agent that is able to escape DNA repair mechanisms; as a result, it exhibits enhanced biological activity and intracellular persistence compared with conventional DNA cross-linking agents (Fig. 1) (Kahl et al. 2019; Hartley 2020).

## Preclinical and clinical studies of anti-CD19 antibodies

### Blinatumomab

Blinatumomab was initially investigated as a treatment for relapsed or refractory B-cell acute lymphoblastic leukaemia (ALL), see Table 1 (Topp et al. 2014, 2015; Kantarjian et al. 2017), and has been approved for this use in both adults and children by the FDA and EMA (US Food and Drug Administration 2021b; European Medical Agency 2021). However, the association of blinatumomab with cytokine release syndrome and neurotoxicity has led to the inclusion of a ‘black box’ warning in the US prescribing information (US Food and Drug Administration 2021b). Blinatumomab has also been formally studied as a treatment for relapsed or refractory NHL (Viardot et al. 2016, 2020; Goebeler et al. 2016).

**Table 1:** Completed clinical trials of blinatumomab in the treatment of B-cell malignancies

Study, NCT number (publication[s])	Phase	Objective(s)	No. of patients /age	Indication	BLI treatment regimen	Comparator	Efficacy outcomes	AEs with BLI^f^		
								Any grade	Grade ≥ 3	AEs of special interest
NCT01209286 Topp et al. (2014)	II	Dose-finding	36/adults	R/R B-ALL	Continuous IVI for 4 wks of 6-wk cycle 5 μg/m^2^/day for 1 wk, then 15 μg/m^2^/day	None	CR/CRh: 69% [MRD: 88%; HSCT: 52%] mOS: 9.8 mo mRFS: 7.6 mo	Pyrexia 81% Fatigue 50% Headache 47% Tremor 36% Leukopenia 19%	Leukopenia 4% Thrombocytopenia 9%	Neuro AEs^a^ 17% G4 CRS 6%
NCT01466179 Topp et al. (2015)	II	Efficacy and safety	189/adults	Ph–R/R B-ALL	Continuous IVI for 4 wks of 6-wk cycle 9 μg/day for the first 7 days, then 28 μg/day for 21 days	None	CR/CRh: 43% [MRD: 82%; HSCT: 40%] mOS: 6.1 mo mRFS: 5.9 mo	Pyrexia 60% Headache 34% Febrile neutropenia 28% Peripheral oedema 26% Nausea 24% Hypokalaemia 24% Constipation 21% Anaemia 20%	Febrile neutropenia 25% Neutropenia 16% Anaemia 14%	G3 neuro AEs 11% G3 CRS 2%
TOWER, NCT02013167 Kantarjian et al. (2017)	III	Efficacy and safety	405/adults	Ph–R/R B-ALL	Induction and consolidation: Continuous IVI for 4 wks of 6-wk cycle 9 μg/day for the first 7 days, then 28 μg/day for 21 days Maintenance: Continuous IVI for 4 wks every 12 wks	Standard CTx (SC)^b^	mOS: BLI: 7.7 mo; SC: 4.0 mo *P* = 0.01 CR/CRh: BLI: 42%; SC: 20% *P* < 0.001	Pyrexia 60% Headache 29% Anaemia 26% Febrile neutropenia 24% Diarrhoea 22% Neutropenia 20% Nausea 19% Thrombocytopenia 18% Hypokalaemia 17%	Febrile neutropenia 21% Neutropenia 18%	≥ G3 neuro AEs 9% ≥ G3 CRS 3%^C^
NCT02393859 Locatelli et al. (2021)	III	Efficacy and safety	108/ < 18 yr	Relapsed B-ALL (consolidation therapy)	Continuous IVI for 4 weeks (1 cycle only) 15 μg/m^2^/day	CTx	EFS:^d^ BLI 69%; chemotherapy 43% *P* < 0.001 ACM: BLI 15%; chemotherapy 30%	Pyrexia 81% Nausea 41% Headache 35% Stomatitis 35% Vomiting 30% Serious AEs: 24% [Chemotherapy: 43%]	Overall: 57.4% [Chemotherapy: 82.4%] Thrombocytopenia 19% Stomatitis 19% Neutropenia 17% Anaemia 15%	Neuro AEs 48% [≥ G3 6%]
NCT02101853 Brown et al. (2021)	III	Efficacy and safety	208/ ≤ 30 yr	Relapsed B-ALL (consolidation therapy pre-HSCT)	Continuous IVI for 4 weeks (two cycles separated by 7-day break) 15 μg/m^2^/day	CTx	2-yr DFS: BLI 54% CTx 39% 2-yr OS: BLI 71% CTx 58%	First cycle: Anaemia 76% WBC decreased 66% ALT increased 64% Fever 53% Neutrophil count decreased 50% Serious AEs:^e^ Infection 15% Febrile neutropenia 5% Sepsis 2% Mucositis 1%	First cycle: Anemia 15% WBC decreased 25% Neutrophil count decreased 33% Lymphocyte count decreased 36%	First cycle: CRS 22% [≥ G3 1%] Encephalopathy 11% [≥ G3 6%] Seizure 4% [≥ G3 1%]

*ACM* all-cause mortality, *AE* adverse event, *ALT* alanine aminotransferase, *B-ALL* B-cell acute lymphoblastic leukaemia, *BLI* blinatumomab, *CR* complete remission/response, *CRh* CR with partial haematological recovery, *CRS* cytokine release syndrome, *CTx* chemotherapy, *DFS* disease-free survival, *EFS* event-free survival, *FLAG* Fludarabine, cytarabine (Ara-C) and Granulocyte colony-stimulating factor, *GX* Grade X, *HSCT* haematopoietic stem cell transplantation, *IVI* intravenous infusion, *mo* months, *(m)OS* (median) overall survival, *MRD* minimal residual disease, *mRFS* median relapse-free survival, *neuro* neurological, *Ph–* Philadelphia chromosome-negative, *R/R* relapsed/refractory, *WBC* white blood cell, *wk* week, *yr* year(s)

^a^Nervous system or psychiatric disorders requiring treatment interruption or permanent discontinuation

^b^Investigator’s choice between FLAG (± anthracycline), high-dose cytarabine, high-dose methotrexate, and clofarabine

^c^CRS of any grade was reported in 14% of blinatumomab recipients

^d^Events: relapse, death, second malignancy or failure to achieve complete remission over a median follow-up period of 22.4 mo

^e^Corresponding rates in the chemotherapy arm were: infection, 65%; febrile neutropenia, 58%; sepsis, 27%; and mucositis, 28%

^f^Selected AEs only. For full details, refer to the original publication

### Non-Hodgkin lymphoma

In a phase I dose-escalation study in 76 heavily pre-treated patients with relapsed or refractory NHL (NCT00274742), the maximum tolerated dose of blinatumomab (administered via continuous intravenous infusion [IVI]) was determined to be 60 μg/m^2^/day (Goebeler et al. 2016). In patients who received this dosage, the overall response rate (ORR) was 69% across NHL subtypes and 55% in patients with DLBCL. However, neurological events were dose-limiting.

In a later dose-finding study in 25 heavily pre-treated patients with DLBCL (NCT01741792), the ORR after one cycle of blinatumomab was 43% (9 of 21 evaluable patients), which included CR in 4 patients. Grade 3 neurological AEs reported in more than one patient were encephalopathy and aphasia (Viardot et al. 2016).

A pooled analysis has been conducted of data from 17 patients from these two trials and from the phase II portion of a third trial (Coyle et al. 2020), all of whom had relapsed or refractory DLBCL and a CR to treatment with blinatumomab (Viardot et al. 2020). Neither the median duration of CR nor median overall survival (OS) were reached after median follow-up times of 15.6 months and 16.4 months, respectively, and the probability of OS at 2 years was estimated to be 77.9%. Fourteen patients had grade ≥ 3 treatment-emergent AEs, and eight patients had serious AEs.

### Tafasitamab

#### Preclinical studies

In vitro/ex vivo and in vivo studies in mice and primates demonstrated the potential of tafasitamab as a therapeutic agent. In an in vitro study of patient-derived B-cell lymphoma and leukaemia lines, tafasitamab substantially increased ADCC relative to an unmodified anti-CD19 antibody, and was also associated with increased ADCP and apoptosis (Horton et al. 2008). In a mouse lymphoma xenograft model, tafasitamab was found to significantly inhibit lymphoma growth, and demonstrated greater antitumour activity than unmodified anti-CD19 (Horton et al. 2008). Tafasitamab also produced rapid, dose-dependent B-cell depletion in blood and lymphoid tissues in cynomolgus monkeys, which have similar immune systems to humans (Zalevsky et al. 2009); a concomitant reduction in NK cells suggested their involvement in the observed B-cell clearance. In contrast, neither B-cell depletion nor reductions in NK cell count were observed with unmodified anti-CD19 antibodies.

In an ex vivo study using cell lines from patients with B-cell chronic lymphocytic leukaemia (CLL), tafasitamab was found to be a significantly more potent inducer of ADCC than either unmodified anti-CD19 antibodies or rituximab (Awan et al. 2010). Additionally, tafasitamab was a moderate inducer of ADCP and had direct cytotoxic effects, but did not activate CDC. This study confirmed that tafasitamab-induced ADCC is mediated by NK cells. Importantly, tafasitamab-induced ADCC was found to be potentiated by the addition of lenalidomide, which stimulates the proliferation and activation of NK cells (Gribben et al. 2015), prompting subsequent clinical investigation into the efficacy of this combination.

The effects of tafasitamab have also been studied in vitro and ex vivo in B-cell ALL (Rafiq et al. 2012; Kellner et al. 2013). In vitro, tafasitamab was found to have potent ADCC-inducing effects and modest direct cytotoxicity, but was superior in these respects to either rituximab or alemtuzumab (anti-CD52) (Rafiq et al. 2012). An ex vivo study, using cell lines from paediatric and adult patients with B-lineage ALL, has also been undertaken to elucidate the Fc-mediated effects of tafasitamab (Kellner et al. 2013). The study confirmed that tafasitamab induces NK cell-mediated ADCC; rituximab and unmodified anti-CD19 antibodies (which were used as controls) were, respectively, ineffective and less potent than tafasitamab.

#### Clinical studies

The first clinical trial of tafasitamab was a phase I dose-escalation study in patients with relapsed or refractory CLL (*n* = 27; Table 2) (Woyach et al. 2014). No maximum tolerated dose was reached, and treatment was generally well tolerated. Preliminary evidence of efficacy was obtained, with 18 patients (66.7%) achieving a response defined using physical examination criteria and laboratory data, and 8 patients (29.6%) achieving a response defined using radiological (i.e. computed tomography) criteria.

**Table 2:** Completed clinical trials of tafasitamab in the treatment of B-cell malignancies

Study, NCT number (publication[s])	Phase	Objective(s)	No. of patients/age	Indication	TAF treatment regimen	Comparator	Efficacy outcomes	AEs with TAF	
								Any grade	Grade ≥ 3
NCT01161511 Woyach et al. (2014)	I	Dose-finding; safety; PK; efficacy	27/adults	R/R CLL	IVI 0.3–12 mg/kg ≤ 9 doses at 3–7-day intervals	None	PR 67% SD 33%^a^ mPFS 199 days	Infusion reaction 67% ALT increase 19% AST increase 15% Fever 15%	Neutropenia 7% Thrombocytopenia 7% Tumour lysis syndrome 4% AST increase 4%
NCT01685008 Jurczak et al. (2019); Jurczak et al. (2018)	IIa	Efficacy and safety	92/adults	R/R NHL	Weekly IVI 12 mg/kg ≤ 3 × 28-day cycles	None	CR 7% ORR 24% 12-mo PFS 35.1%	Infusion reaction 12% Neutropenia 12%	Neutropenia 10% Thrombocytopenia 4% Anaemia 3% Pneumonia 3%
L-MIND, NCT02399085 Salles et al. (2020); Duell et al. (2021)	II	Efficacy and safety	81/adults	R/R DLBCL	Weekly IVI 12 mg/kg (cycles 1–3) Fortnightly IVI 12 mg/kg (cycle 4 onwards) ICW lenalidomide PO 25 mg/day (days 1–21 of each 28-day cycle)	None	ORR 57.5% CR 40% PR 17.5% mPFS 11.6 mo mDoR 43.9 mo mOS 33.5	Neutropenia 49% Serious AEs 51%	Neutropenia 48% Thrombocytopenia 17% Febrile neutropenia 12%

*AE* adverse event, *ALT* alanine aminotransferase, *AST* aspartate aminotransferase, *CLL* chronic lymphocytic leukaemia, *CR* complete remission/response, *DLBCL* diffuse large B-cell lymphoma, *ICW* in combination with, *IVI* intravenous infusion, *mo* months, *(m)OS* (median) overall survival, *(m)PFS* (median) progression-free survival, *NHL* non-Hodgkin lymphoma, *ORR* overall/objective response rate, *PK* pharmacokinetics, *PO* per oral/orally, *PR* partial response, *R/R* relapsed/refractory, *SD* stable disease, *TAF* tafasitamab

^a^On the basis of physical examination and laboratory studies

^b^After a median follow-up of 19.6 months, 36% of patients had died. Survival rates at 12 and 18 months were 74% and 64%, respectively

A phase IIa trial in patients with relapsed or refractory NHL was initiated following the successful long-term use of salvage/maintenance treatment with tafasitamab monotherapy in a patient with DLBCL who had early relapse following standard first- and second-line treatment (Jurczak et al. 2016, 2018, 2019). In this open-label, multicentre, single-arm study, 92 patients received up to three 28-day cycles of tafasitamab as monotherapy (12 mg/kg per week by IVI) (Jurczak et al. 2019). The primary efficacy endpoint was investigator-assessed ORR, which was achieved in 23.9% (95% CI 15.6–33.9). The median duration of response was 24.0 months (95% CI 11.1–not reached [NR]) overall, and 20.1 months (95% CI 1.1–NR) in the 35 patients with DLBCL; treatment was well tolerated, with most treatment-emergent AEs being of mild intensity. Interestingly, and consistent with the role of tafasitamab–NK cells interactions, median progression-free survival (PFS) was at least twice as long in patients with NK cell counts > 100 cells/μL at baseline compared with those with counts < 100 cells/μL: 4.2 versus 2.1 months, respectively, in patients with DLBCL, and 8.8 versus 3.2 months in patients with follicular lymphoma.

The first results of the phase II L-MIND trial, an open-label, single-arm study of tafasitamab in combination with lenalidomide in adults (*n* = 81) with relapsed or refractory DLBCL who were ineligible for high-dose chemotherapy and subsequent ASCT, were reported in 2020 (Salles et al. 2020). Tafasitamab (12 mg/kg) was administered intravenously over 2 h every week (cycles 1–3) or every 2 weeks (cycle 4 onwards), and oral lenalidomide (starting dose 25 mg) was given daily on days 1–21 of each 28-day cycle. Treatment was continued for up to 12 cycles, and was followed by tafasitamab monotherapy until disease progression occurred. Recently, the results of the 3-year follow-up of the L-MIND clinical trial have been published (Duell et al. 2021). After ≥ 35 months’ follow-up, ORR was 57.5%, including a complete response in 40.0% of patients and a partial response in 17.5% of patients. Median duration of response (DoR) was 43.9 months, median OS was 33.5 months, and median PFS was 11.6 months (Duell et al. 2021). The combination of tafasitamab and lenalidomide was well tolerated, with most non-haematological AEs being of grade 1 or 2 intensity.

The proportion of patients with CR was notable because relapsed or refractory DLBCL is difficult to treat and has a poor prognosis. Thus, the L-MIND trial provides clinical evidence that, as suggested by earlier ex vivo experiments, tafasitamab/lenalidomide is a synergistic combination in which the affinity of tafasitamab for both effector and targets cells is magnified by the immunomodulating effects of lenalidomide (such as stimulation of NK cell proliferation, as well as activation and enhancement of NK-mediated ADCC) (Awan et al. 2010; Gribben et al. 2015). In May 2020, the EMA CHMP validated the application for marketing authorization. In July 2020, the US FDA granted accelerated approval. In June 2021, the CHMP recommended the use of tafasitamab and the application is now being reviewed by the European Commission which has the authority to grant marketing authorization in the EU.

To assess the contribution of tafasitamab to the clinical effects of tafasitamab/lenalidomide combination therapy, outcomes in 76 real-world patients treated with lenalidomide monotherapy (the ‘RE-MIND’ cohort) were retrospectively compared with those in 76 propensity score-matched participants from the L-MIND trial (the ‘L-MIND’ cohort) (Nowakowski et al. 2020; Zinzani et al. 2021). Patients in the L-MIND cohort were significantly more likely to respond than those in the RE-MIND cohort (ORR 67.1% vs 34.2%; odds ratio 3.89; 95% CI 1.90–8.14; *P* < 0.0001 investigator assessment) and had significantly longer OS (median not reached vs 9.3 months; 95% CI 5.0–15.3; *P* = 0.0008), indicating that tafasitamab and lenalidomide may have synergistic effects in vivo. A second study, RE-MIND2 (NCT04697160), is under way, and will compare outcomes from the L-MIND study with those from matched real-world patients treated with guideline-recommended salvage regimens such as R-GEMOX (rituximab, gemcitabine and oxaliplatin) and BR (bendamustine and rituximab).

A number of clinical trials of tafasitamab are also planned or are currently ongoing (Table 3). The InMIND trial (NCT04680052) is a phase III study of tafasitamab versus placebo, both in combination with lenalidomide and rituximab, in patients with relapsed or refractory follicular lymphoma or marginal zone lymphoma. The phase I First-MIND trial assesses the safety and preliminary efficacy of tafasitamab (with or without lenalidomide) in combination with R-CHOP in newly diagnosed DLCBL (NCT04134936). A large phase III trial in newly diagnosed DLBCL, frontMIND, is recruiting, and will investigate the efficacy and safety of tafasitamab/lenalidomide, added to R-CHOP vs R-CHOP, in high-risk and high-intermediate risk patients (NCT04824092). Lastly, the phase Ib/IIa topMIND trial will evaluate the safety, pharmacokinetics and efficacy of tafasitamab in combination with the phosphatidylinositol 3-kinase δ (PI3Kδ) inhibitor parsaclisib in adults with NHL or CLL (NCT04809467).

**Table 3:** Ongoing clinical trials of tafasitamab and loncastuximab tesirine in B-cell malignancies

Study, NCT number	Design	Estimated enrollment	Outcome(s)	Study population	Objective
Tafasitamab					
RE-MIND2, NCT04697160	Observational, retrospective cohort study	3729	OS, ORR, CRR, DoR, EFS, PFS, discontinuation due to AEs, duration of treatment exposure	Adults with R/R DLBCL	To compare outcomes of the L-MIND cohort with patients receiving guideline-recommended salvage regimens, such as R-GEMOX
InMIND, NCT04680052	Phase III, randomized, double-blind, placebo-controlled, multicentre study	618	PFS, CRR, MRD-negativity rate, OS, DoR, TEAEs	Adults with follicular lymphoma or marginal zone lymphoma	To determine if TAF + lenalidomide + rituximab provides improved clinical benefit vs lenalidomide + rituximab
First-MIND, NCT04134936	Phase Ib, randomized, open-label study	60	TEAEs, ORR, CRR, AEs, ORR, PFS, EFS, OS	Adults with DLBCL	To evaluate the safety and preliminary efficacy of TAF + R-CHOP vs TAF + lenalidomide + R-CHOP
frontMIND, NCT04824092	Phase III, randomized, double-blind, placebo-controlled, multicenter study	880	PFS, EFS, OS, CR, OS, ORR	Previously untreated, high-intermediate and high-risk adults with newly diagnosed DLBCL	To evaluate the efficacy and safety TAF + lenalidomide + R-CHOP vs R-CHOP
topMIND, NCT04809467	Phase Ib/IIa, open-label, single-arm,	100	TEAEs, DLTs, ORR, PK	Adults with R/R NHL or CLL	To evaluate if TAF + parsaclisib can be safely combined at the recommended Phase II dose and dosing regimen previously established for each compound (when used as a treatment option for adult participants with R/R B-cell malignancies)
Loncastuximab tesirine					
LOTIS-3, NCT03684694	Phase I/II, open-label study	161	AEs, serious AEs, DLTs, dose reductions, change in vital signs or ECOG PS, CRR	Adults with DLBCL or MCL	To determine the safety and efficacy LON + ibrutinib

*AE* adverse event, *CLL* chronic lymphocytic leukemia, *CRR* complete response rate, *DLBCL* diffuse large B-cell lymphoma, *DLT* dose-limiting toxicities, *DoR* duration of response, *ECOG PS* Eastern Cooperative Oncology Group performance status, *EFS* event-free survival, *LON* loncastuximab tesirine, *MCL* mantle cell lymphoma, *MRD* minimal residual disease, *NHL* non-Hodgkin lymphoma, *ORR* overall/objective response rate, *PFS* progression-free survival, *PK* pharmacokinetics, *R-CHOP* rituximab, cyclophosphamide, doxorubicin, vincristine and prednisone, *R-GEMOX* rituximab, gemcitabine and oxaliplatin, *R/R* relapsed/refractory, *TAF* tafasitamab, *TEAE* treatment-emergent adverse event

## Loncastuximab tesirine

In human cell lines expressing CD19, loncastuximab tesirine demonstrated potent, selective cytotoxicity (Zammarchi et al. 2018). Additionally, in models of subcutaneous and disseminated human tumours, loncastuximab tesirine exhibited potent, dose-dependent antitumour activity that was superior to that of antibody–drug conjugates delivering tubulin inhibitors (Zammarchi et al. 2018). Further preclinical studies showed that loncastuximab tesirine was pharmacokinetically stable and had good tolerability in mice and cynomolgus monkeys.

These findings led to the design of a phase I dose-escalation and dose-expansion study of loncastuximab tesirine in adults with relapsed or refractory NHL (NCT02669017; Table 4). In the dose-escalation phase (*n* = 88), doses ranging from 15 to 200 μg/kg were administered intravenously once every 3 weeks until disease progression or patient withdrawal from the study (Kahl et al. 2019). At doses of 120 μg/kg and above, the ORR was 59.4% (40.6% CR, 18.8% PR), for all doses combined, median OS was 11.6 months, median PFS was 4.8 months and median duration of response 5.5 months. Most patients experienced AEs; grade ≥ 3 fatigue, dyspnoea and abnormalities of haematological or liver function were reported in ≥ 5% of patients (Kahl et al. 2019). In the final analysis of 183 patients, the ORR was 45.6%, with 26.7% achieving CR (Hamadani et al. 2021). Although the maximum tolerated dose was not reached, cumulative toxicity was higher at 200 μg/kg (Hamadani et al. 2021).

**Table 4:** Completed clinical trials of loncastuximab tesirine in the treatment of B-cell malignancies

Study, NCT number (publication [s])	Phase	Objective(s)	No. of patients/age	Indication	LON treatment regimen	Comparator	Efficacy outcomes	AEs with LON	
								Any grade	Grade ≥ 3
NCT02669017 Kahl et al. (2019)	I	Dose escalation	88 / adults	R/R NHL	IVI 15–200 μg/kg q3w	None	At ≥ 120 μg/kg: ORR 59% CR 41% All patients: mOS 11.6 mo mPFS 4.8 mo	All doses: Platelet count decreased 74% Neutrophil count decreased 62.5% Fatigue 49% Peripheral oedema 35% GGT increased 33% Nausea 32% Dyspnoea 20%	All doses: Neutrophil count decreased 41% Platelet count decreased 28% GGT increased 19% Anaemia 12.5% Dyspnoea 6%
NCT02669017 Hamadani et al. (2021)	I	Dose escalation and expansion	183 / adults	R/R NHL	Escalation: IVI 15–200 μg/kg q3w Expansion: 120 and 150 μg/kg q3w	None	ORR 46% CR 27% mOS 8.3 mo mPFS 3.1 mo	All doses: Platelet count decreased 71% Neutrophil count decreased 59% Anaemia 33% Fatigue 43% Peripheral oedema 32% GGT increased 31% Nausea 32% Rash 25% Dyspnoea 22%	All doses: Neutrophil count decreased 40% Platelet count decreased 27% GGT increased 21% Anaemia 15%
LOTIS-2, NCT03589469 Caimi et al. (2021)	II	Efficacy and safety	145 / adults	R/R DLBCL	IVI on day 1 of each 21-day cycle Cycles 1 and 2: 150 μg/kg Cycle 3 onwards: 75 μg/kg	None	ORR 48% CR 24% mOS 9.9 mo mPFS 4.9 mo mRFS 13.4 mo	GGT increased 41% Neutropenia 39% Thrombocytopenia 33% Fatigue 28%	Neutropenia 26% Thrombocytopenia 18% GGT increased 17%
NCT02669264 Jain et al. (2020)	I	Safety	35 / adults	R/R B-ALL	IVI 15–150 μg/kg q3w or 50 μg/kg q1w	None	Not formally assessed	Nausea 43% Febrile neutropenia 37% AST increased 31% GGT increased 29%	Febrile neutropenia 29% AST increased 17% Blood bilirubin increased 14% GGT increased 14%

*AE* adverse event, *AST* aspartate aminotransferase, *B-ALL* B-cell acute lymphoblastic leukaemia, *CR* complete remission/response, *DLBCL* diffuse large B-cell lymphoma, *GGT* gamma-glutamyl transferase, *IVI* intravenous infusion, *LON* loncastuximab tesirine, *mo* months, *mOS* median overall survival, *mPFS* median progression-free survival, *mRFS* median relapse-free survival, *NHL* non-Hodgkin lymphoma, *ORR* overall/objective response rate, *q*x*w* every x weeks, *R/R* relapsed/refractory

Based on these results, a phase II, single-arm trial of loncastuximab tesirine monotherapy (LOTIS-2; NCT03589469), using a starting dose of 150 μg/kg, was initiated in heavily pre-treated patients with relapsed or refractory DLBCL (Table 4) (Caimi et al. 2021). In this trial, the ORR was 48.3%, with equal numbers of patients achieving CR and PR. Median OS and PFS were 9.9 and 4.9 months, respectively (Caimi et al. 2021). In April 2021, the FDA-approved loncastuximab tesirine for the treatment of relapsed or refractory large B-cell lymphomas after 2 or more lines of treatment, including DLBCL (US Food and Drug Administration 2021a).

Ongoing clinical trials include LOTIS-3 (NCT03684694), a two-part dose-escalation and dose-expansion trial of loncastuximab tesirine (in combination with the Bruton’s tyrosine kinase inhibitor ibrutinib, at a dosage of 560 mg/day) in patients with relapsed or refractory DLBCL or mantle cell lymphoma (Table 3). In an interim analysis of the first part of the trial (*n* = 25), the maximum tolerated dose of loncastuximab tesirine for use in combination with ibrutinib was determined to be 60 μg/kg; at this dosage, seven of the 12 (58.3%) patients who were evaluable for response had CR, and a further two patients had PR, giving an ORR of 75.0% (Depaus et al. 2020). The second part of the LOTIS-3 trial is ongoing.

Loncastuximab tesirine has also undergone preliminary assessment as a treatment for relapsed or refractory B-cell ALL (Table 4) (Jain et al. 2020). A phase I, open-label, single-arm, multicentre study was conducted in 35 patients to evaluate the safety, tolerability and immunogenicity of loncastuximab tesirine 15–150 μg/kg, and to determine its maximum tolerated dose and pharmacokinetic profile (NCT02669264). The maximum tolerated dose was not reached, and anti-drug antibodies were not detected at clinically relevant levels.

## Place in therapy

Immunotherapy with monoclonal antibodies (e.g. with the anti-CD20 agent rituximab) has revolutionized treatment in patients with B-cell malignancies. However, treatment options for patients who do not respond to, or have early relapse following, first-line rituximab-based treatment have long been very limited. The recent development of three novel monoclonal antibodies that target the B-cell surface antigen CD19, each of which belongs to a distinct molecular class, will expand the available options for patients with relapsed or refractory disease.

Blinatumomab has been most extensively studied in relapsed or refractory B-cell ALL, and is licensed for this indication in Europe and the US in both adults and children. However, cytokine release syndrome and neurotoxicity, as well as the need for continuous IVI over 4 weeks of each 6-week cycle may limit its clinical usefulness. Less is known about its efficacy and safety in relapsed or refractory NHL. Tafasitamab, on the other hand, has been extensively studied in patients with relapsed or refractory DLBCL, and in the US is approved for use in this indication in combination with lenalidomide in adults who are ineligible for ASCT. Tafasitamab is being studied as part of a first-line treatment regimen for DLBCL, and in the relapsed/refractory setting in other forms of B-cell NHL. Like tafasitamab, loncastuximab tesirine has been studied in heavily pre-treated patients with DLBCL, but there is less experience with its use. Superficially, outcomes appear comparable between the three agents, although long-term follow-up is required to determine the duration of response with some certainty.

Anti-CD19 antibodies have major advantages over CAR T-cell therapy in that they are more readily available and can be used in patients with rapidly evolving disease. Moreover, they are relatively easy to use, as there is now a high level of expertise with monoclonal antibodies in many cancer centres. Monoclonal antibodies are also considerably less expensive and resource-intensive than CAR T-cell therapy, and, additionally, may be safer in older, frailer patients who cannot tolerate high-dose chemotherapy. Anti-CD19 antibodies may also be a useful option in the event of non-response or relapse following CAR T-cell therapy; to date, however, no formal clinical trials have been performed to support their use in this setting.

The availability of several new therapeutic options for relapsed or refractory B-cell malignancies raises the question as to the best sequence in which to use them for maximal clinical benefit. Further controlled studies of sufficiently long duration are needed to determine whether the order in which treatments are used influences OS or other key outcomes.

## Conclusions

Blinatumomab, tafasitamab and loncastuximab tesirine are novel monoclonal antibody-based treatments that target CD19, a cell surface protein that is present throughout the B-cell maturation process, and consequently is an important therapeutic target in patients with B-cell malignancies. In particular, the combination of tafasitamab and lenalidomide is associated with synergistic interactions between the two agents and appears to have good activity against B-cell NHLs. This combination has been extensively studied in patients with relapsed or refractory DLBCL, and should be considered, together with loncastuximab tesirine, as a valid alternative to CAR T-cell therapy in patients who are ineligible for ASCT.

## Data Availability

Not applicable.

## Footnotes

1 FcγRIIIa is also known as CD16a**.**
